# Multi-instance learning with attention mechanism for coronary artery stenosis detection on coronary computed tomography angiography

**DOI:** 10.1093/ehjdh/ztaf029

**Published:** 2025-04-01

**Authors:** Vibha Gupta, Petur Petursson, Lukas Hilgendorf, Aidin Rawshani, Jan Borén, Truls Råmunddal, Elmir Omerovic, Antros Louca, Oskar Angerås, Justin Schneiderman, Kristofer Skoglund, Deepak L Bhatt, Magnus Kjellberg, Erik Andersson, Carlo Pirazzi, Araz Rawshani

**Affiliations:** Department of Molecular and Clinical Medicine, Institute of Medicine, University of Gothenburg, Bruna stråket 16, 413 45 Göteborg, Gothenburg, Sweden; Wallenberg Center for Molecular and Translational Medicine, Wallenberg Laboratory, Blå stråket 5, staircase H, Sahlgrenska University Hospital, 413 45 Gothenburg, Sweden; Centre for Digital Health, Område Digitalisering, Sahlgrenska University Hospital, Blå Stråket 5, SE-413 45 Gothenburg, Sweden; Department of Thoracic Surgery and Cardiology, Sahlgrenska University Hospital, Blå Stråket 5, SE-413 45 Gothenburg, Västra Götaland, Sweden; Department of Molecular and Clinical Medicine, Institute of Medicine, University of Gothenburg, Bruna stråket 16, 413 45 Göteborg, Gothenburg, Sweden; Centre for Digital Health, Område Digitalisering, Sahlgrenska University Hospital, Blå Stråket 5, SE-413 45 Gothenburg, Sweden; Department of Molecular and Clinical Medicine, Institute of Medicine, University of Gothenburg, Bruna stråket 16, 413 45 Göteborg, Gothenburg, Sweden; Department of Molecular and Clinical Medicine, Institute of Medicine, University of Gothenburg, Bruna stråket 16, 413 45 Göteborg, Gothenburg, Sweden; Department of Thoracic Surgery and Cardiology, Sahlgrenska University Hospital, Blå Stråket 5, SE-413 45 Gothenburg, Västra Götaland, Sweden; Department of Thoracic Surgery and Cardiology, Sahlgrenska University Hospital, Blå Stråket 5, SE-413 45 Gothenburg, Västra Götaland, Sweden; Department of Molecular and Clinical Medicine, Institute of Medicine, University of Gothenburg, Bruna stråket 16, 413 45 Göteborg, Gothenburg, Sweden; Wallenberg Center for Molecular and Translational Medicine, Wallenberg Laboratory, Blå stråket 5, staircase H, Sahlgrenska University Hospital, 413 45 Gothenburg, Sweden; Department of Molecular and Clinical Medicine, Institute of Medicine, University of Gothenburg, Bruna stråket 16, 413 45 Göteborg, Gothenburg, Sweden; Wallenberg Center for Molecular and Translational Medicine, Wallenberg Laboratory, Blå stråket 5, staircase H, Sahlgrenska University Hospital, 413 45 Gothenburg, Sweden; Department of Thoracic Surgery and Cardiology, Sahlgrenska University Hospital, Blå Stråket 5, SE-413 45 Gothenburg, Västra Götaland, Sweden; Institute of Neuroscience and Physiology, University of Gothenburg, Medicinaregatan 11, Box 430, SE-405 30 Gothenburg, Sweden; Department of Molecular and Clinical Medicine, Institute of Medicine, University of Gothenburg, Bruna stråket 16, 413 45 Göteborg, Gothenburg, Sweden; Department of Thoracic Surgery and Cardiology, Sahlgrenska University Hospital, Blå Stråket 5, SE-413 45 Gothenburg, Västra Götaland, Sweden; Mount Sinai Fuster Heart Hospital, Icahn School of Medicine at Mount Sinai, One Gustave L. Levy Place, New York, NY 10029, USA; AI ​​Competence Center, Sahlgrenska University Hospital, Blå Stråket 5, SE-413 45 Gothenburg, Sweden; Department of Molecular and Clinical Medicine, Institute of Medicine, University of Gothenburg, Bruna stråket 16, 413 45 Göteborg, Gothenburg, Sweden; Wallenberg Center for Molecular and Translational Medicine, Wallenberg Laboratory, Blå stråket 5, staircase H, Sahlgrenska University Hospital, 413 45 Gothenburg, Sweden; Centre for Digital Health, Område Digitalisering, Sahlgrenska University Hospital, Blå Stråket 5, SE-413 45 Gothenburg, Sweden; Department of Thoracic Surgery and Cardiology, Sahlgrenska University Hospital, Blå Stråket 5, SE-413 45 Gothenburg, Västra Götaland, Sweden; Department of Molecular and Clinical Medicine, Institute of Medicine, University of Gothenburg, Bruna stråket 16, 413 45 Göteborg, Gothenburg, Sweden; Wallenberg Center for Molecular and Translational Medicine, Wallenberg Laboratory, Blå stråket 5, staircase H, Sahlgrenska University Hospital, 413 45 Gothenburg, Sweden; Centre for Digital Health, Område Digitalisering, Sahlgrenska University Hospital, Blå Stråket 5, SE-413 45 Gothenburg, Sweden; Department of Thoracic Surgery and Cardiology, Sahlgrenska University Hospital, Blå Stråket 5, SE-413 45 Gothenburg, Västra Götaland, Sweden

**Keywords:** Coronary artery disease (CAD), Deep learning, Multi-instance learning, Attention scores, Curved multiplanar reformations (CMRs), Stenosis

## Abstract

**Aims:**

Accurate detection of coronary artery stenosis (CAS) on coronary computed tomography angiography is vital for saving lives, as timely diagnosis can prevent severe cardiac events. However, this task remains challenging due to data complexity and variability in imaging protocols. Deep learning offers promising potential to automate detection, but robust methods are essential to address real-world challenges effectively and enhance patient outcomes.

**Methods and results:**

A total of 900 cases with curved multiplanar reformations, pre-generated during routine clinical workflows, were used to train a multi-instance learning (MIL) model for detecting significant CAS (≥50% luminal obstruction) in the left anterior descending (LAD), right coronary artery (RCA), and left circumflex (LCX), comprising 776 LAD, 694 RCA, and 600 LCX reconstructions. Patient-level predictions utilized attention scores to quantify each slice’s contribution, ensuring a robust and interpretable diagnostic approach. The model achieved the best performance for LAD [area under the curve (AUC): 0.92, 95% confidence interval (CI): 0.87–0.96; Brier score: 0.11], followed by RCA (AUC: 0.91, 95% CI: 0.82–0.999; Brier score: 0.09) and LCX (AUC: 0.92, 95% CI: 0.84–0.99; Brier score: 0.07). Calibration was good for LAD but less precise for RCA and LCX. Attention scores enhanced diagnostic precision by focusing on the most relevant slices.

**Conclusion:**

This study highlights the potential of MIL models for CAS detection, with remarkable performance in the LAD. By using attention scores, the model effectively identifies key slices from real-world data, seamlessly integrating with routine clinical workflows. Multi-range pre-processing addresses data complexity, enhancing diagnostic accuracy and supporting clinical decision-making.

## Graphical Abstract

**Graphical Abstract:**
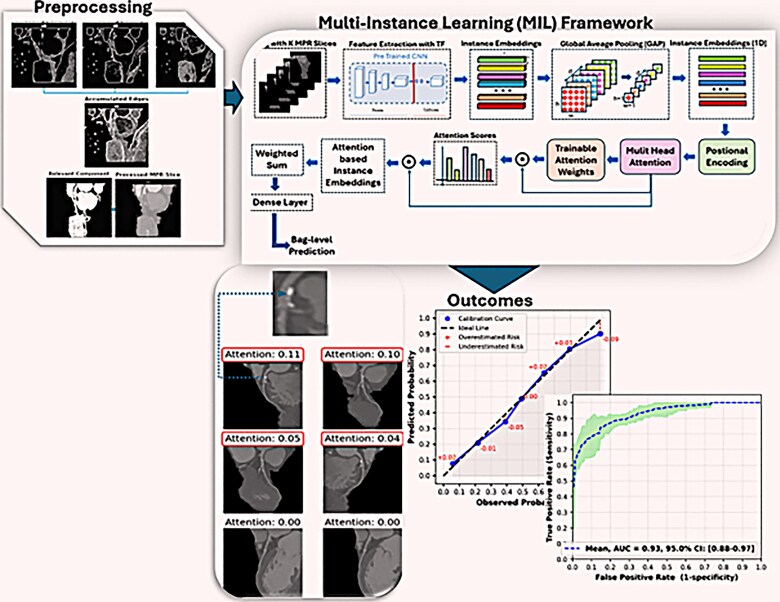

## Introduction

Coronary artery disease (CAD) remains the leading cause of death worldwide.^1^ The growing availability and advancements in coronary computed tomography angiography (CCTA) can significantly improve the detection and characterization of atherosclerotic plaque and their features.^2–5^ However, interpreting CCTA studies is a complex, time-intensive process that demands substantial expertise, limiting capacity. Deep learning has the potential to revolutionize this task, by either augmenting expert workflows or fully automating the interpretation.^6–10^ The use of end-to-end deep learning, i.e. models trained on raw, unprocessed, input data and labels, represents an avenue to leveraging large-scale databases in cardiovascular imaging.^11^

Gulati *et al*.^12^ recommend CCTA as a potential initial test for evaluating patients with chest pain, as outlined in their guidelines for the evaluation and diagnosis of chest pain. In addition, the Society of Cardiovascular Computed Tomography recommends various imaging techniques for coronary artery assessment, including axial images, curved multiplanar reformations (CMRs), and maximum intensity projections.^13,14^ Curved multiplanar reformation images allow visualization of the entire length of the artery in any desired cross-section, facilitating detailed assessment along the vessel. Despite these advances, automated methods for analysing CMR images face significant challenges due to the variability in disease presentation across slices and the limited availability of detailed slice-level annotations, which are crucial for training conventional machine learning models. This variability complicates the development of robust automated systems, requiring pre-processing pipelines that can standardize inputs across diverse data sets to ensure consistent model performance. These challenges underscore the need for innovative approaches that can effectively handle both the imaging protocol variations and the limitations of slice-level data availability.

To address these limitations, this study employs multi-instance learning (MIL), a framework well suited for handling data with sparse or weak labels.^15–17^ Unlike conventional methods that assign uniform labels to all slices or treat them independently, MIL processes patient data as a ‘bag of slices’ to make patient-level predictions. This approach captures the spatial variability of disease manifestations across slices, enabling the model to focus on the most relevant regions for diagnosis. Multi-instance learning enhances the understanding of stenosis detection and supports practical clinical needs by streamlining the diagnostic process. This approach may reduce the need for clinicians to examine every slice, potentially facilitating rapid and accurate interpretation, which is crucial for patient management and treatment decisions. Additionally, the study incorporates a multi-range pre-processing pipeline, which addresses the challenges posed by imaging variability and ensures consistent model performance across diverse data sets.

Although MIL has been widely applied in pathology, such as analysing whole-slide images to identify malignancies, its application in CAD detection remains limited.^18,19^ By integrating spatial context, attention mechanisms, and incorporating multiple views of anatomical structures, this study bridges critical gaps in existing methods. The proposed framework reduces blind spots in CCTA scans, enhances spatial connectivity, and offers a robust, interpretable solution for improving diagnostic precision in CAD detection.

## Methods

The study was approved by the Swedish Ethical Review Authority (no. 2023-03311-02).

### Patient selection

The original data set comprised all CCTA examinations (6293) performed between 2010 and 2021 in Västra Götaland County, Sweden. This data set included consecutive patients of all ages, men and women, who underwent contrast-enhanced, ECG-gated CCTA as part of their clinical care. All studies were interpreted by accredited radiologists, following established protocols for scanner use, administration of beta-blockers, and sublingual glyceryl nitrate. The vast majority of these cases were referred for coronary artery evaluation. We did not include studies that did not evaluate the coronary arteries. To ensure the model’s applicability across the spectrum of patients, we did not exclude any studies based on the cause for referral. The sole inclusion criterion was the completion of a CCTA with expert interpretation of the coronary arteries. About 93% of all CCTA cases were expert evaluated. Supplementary material online, *Figure S1A* provides a flowchart illustrating the patient recruitment process, including the selection criteria and the flow of participants into the study.

### Multiplanar reformations

We included all available CMR images (slices) generated by radiologists as part of the routine clinical process. No additional CMR images were created specifically for this study. To work with an MIL framework, we designed the data into ‘bags,’ each containing 36 multiplanar reformation (MPR) slices, a standard practice throughout the majority of the study period. For patients with more than 36 images, the data set was divided into multiple 36-image bags. Conversely, for patients with fewer than 36 images, we replicated existing images to meet the required 36 per bag. This handling also applied to cases where the number of images exceeded 36, resulting in an additional, incomplete bag being created with replicated images to ensure the 36-image requirement was met. This padding and filling approach ensured that all patients were considered for analysis, regardless of the number of slices they had. To avoid any overlap between training, validation, and testing data sets, patients with multiple image bags were exclusively assigned to one data set (either training, validation, or testing) to prevent data leakage.

### Label extraction and stenosis severity task

Labels for the supervised model were obtained from the radiologist’s final report, which included information on stenoses, plaque types, calcium scores, and other relevant findings. A physician trained in CCTA (Level 1 training) classified, using the radiologist’s written evaluation, the degree of stenosis in coronary artery segments 1 through 15, with the possibility of consulting an experienced interventional cardiologist for second opinions in ambiguous cases. Stenosis was graded using the following cut-offs: 0% (no stenosis), 1–24% (minimal), 25–49% (mild), 50–69% (moderate), 70–99% (severe), and 100% (total occlusion).

For modelling purposes, the highest level of stenosis observed in any segment was assigned as the artery-level label, specifically focusing on major coronary arteries left anterior descending (LAD), right coronary artery (RCA), and left circumflex (LCX). The binary classification task was designed to identify cases with moderate or greater stenosis (50% or more).

### Pre-processing pipeline

During the pre-processing of CMR slices, we aimed to preserve both anatomical and pathological structures without compressing the attenuation values into overly narrow Hounsfield unit (HU) ranges. However, relying on a single attenuation window posed challenges in visualizing certain structures, such as soft plaques and distal artery segments, which are often not fully captured within a single intensity range. To address this limitation, we implemented a multi-attenuation range approach, consistent with the practice of multiple-level thresholding, which is commonly used for segmentation purposes in various fields. The segmentation of the HU range into multiple intervals captures diverse features across these ranges. These intervals were identified based on expert input, ensuring clinical relevance as certain structures are usually found in similar attenuation ranges. The chosen ranges were as follows: (−150, −50), (−50, 50), (50, 130), (130, 400), and (400, 1000). This approach allows us to capture a comprehensive spectrum of features, including soft plaques, vessel walls, and calcifications, which are often overlooked when using a single intensity range.

Data within each intensity range were clipped, normalized, and processed with Sobel edge detection to emphasize structural boundaries. The strongest edges from all intensity ranges were then merged using a maximum response function, enhancing the visibility of critical anatomical and pathological structures. A threshold was then applied to remove irrelevant low-attenuation regions. A small object removal filter was used to discard unnecessary structures, such as objects smaller than 20 pixels. This process reduces noise while preserving prominent features such as vessel boundaries and plaques.

**Figure 1:**
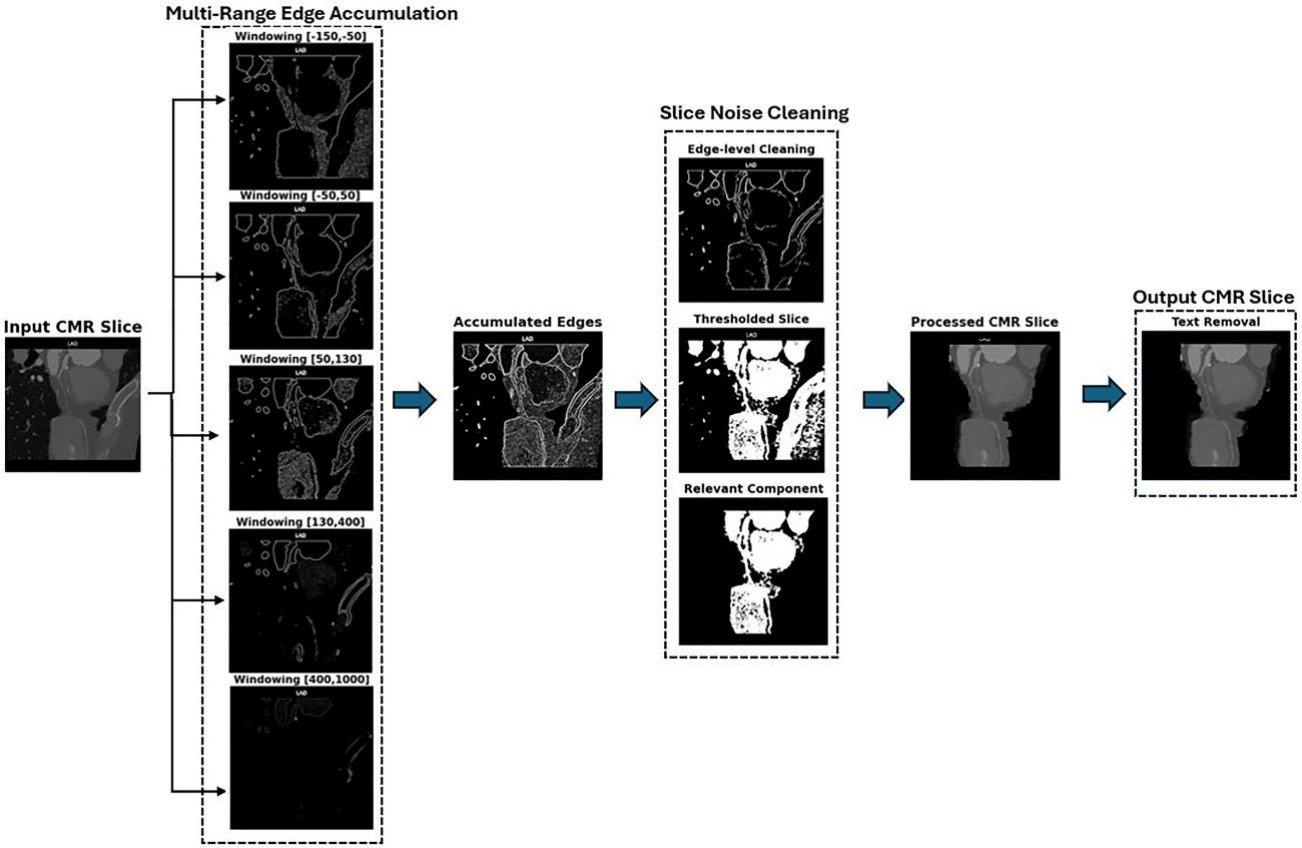
The pre-processing pipeline, demonstrating the visual output at each stage, including multi-windowing, edge extraction, noise, and text removal.

Finally, connected component labelling was employed to isolate arterial structures, with regions ranked based on metrics such as area, major axis length, and perimeter. This ensured that the largest and most relevant regions were retained, resulting in images that are cleaner and more precisely localized. This ensured that the largest and most relevant regions were retained, resulting in clean, focused, and precise images for analysis. Supplementary material online, *Figure S1B* outlines the pipeline from data acquisition to prediction. *Figure 1*, in contrast, illustrates the pre-processing, showcasing the visual output at each stage of the process. The pre-processing pipeline was a fully automated process, utilizing predefined attenuation ranges adjusted according to clinical relevance. This pipeline not only enhances the visual representation of soft plaques and arterial walls but also effectively reduces unwanted structures that may affect the analysis of stenosis in arteries.

### Machine learning framework and evaluation

**Figure 2:**
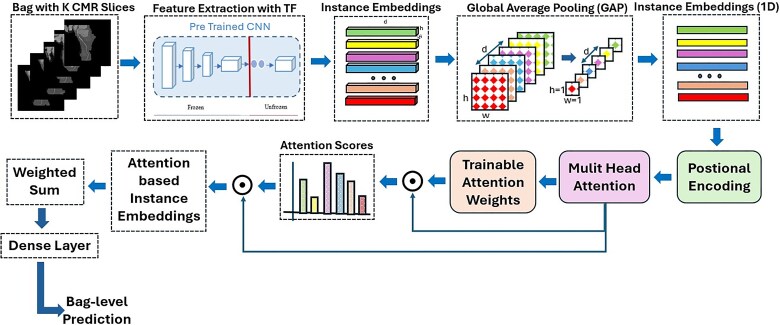
Proposed framework for stenosis detection, detailing each stage from multiplanar reformations slices (Bag) input to the final prediction, incorporating positional encoding, multi-head attention, and the multi-instance learning framework.

The machine learning framework is illustrated in *Figure 2*. Multi-instance learning effectively tackles the challenge of weak supervision, where only patient-level labels are available, but individual slice-level annotations are missing. This method aligns with the clinical assumption that in a sufficiently large set of CMR images, at least one slice will reveal features indicative of arterial narrowing, should they be present. Furthermore, it emphasizes the importance of analysing multiple views of the same anatomical structures to accurately assess the degree of stenosis. Multi-instance learning allows for the diverse perspectives across the slices to be effectively utilized.

Positional encoding plays a critical role in this context by preserving the spatial relationships among the slices. Curved multiplanar reformation slices capture similar anatomical information from different angles, requiring a model that can recognize how these views relate to each other. By encoding the positional context, the model is better equipped to leverage the similarities across slices while accounting for their differing perspectives. The multi-head attention (MHA) mechanism enhances the model’s ability to discern which slices contribute most significantly to the diagnosis, providing a solution to the challenges posed by weak labelling.

In our framework, an attention mechanism is used to assign a weight to each image instance within a patient’s data bag. This weight is represented by an attention score, a learned scalar that reflects the image’s diagnostic relevance. These attention scores serve two key purposes: first, they enhance diagnostic performance by focusing on the most informative regions of the images and second, they improve the model’s interpretability by identifying which images have the most influence on the final prediction.

We utilized transfer learning from the VGG16 base architecture, initialized with ImageNet weights. Key hyperparameters were optimized, including a learning rate of 0.0001, dropout rate of 0.2, and L2 (0.0001) regularization. The first 15 layers of VGG16 were frozen to retain pre-trained features, and MHA was applied to improve feature extraction, with four attention heads used to focus on the most relevant image features.

The framework was trained using the binary cross-entropy loss function, and an ADAM optimizer. Early stopping with a patience of 7 epochs and learning rate reduction on plateau (factor 0.2, patience of 5 epochs) were employed to fine-tune the model and prevent overfitting. To address the class imbalance between positive and negative cases, class weighting was applied during training, along with data augmentation techniques (such as zoom and shift but excluding rotation due to concerns that it might affect the positional encoding) to further prevent overfitting.

For model training, validation, and evaluation, the data set was randomly divided into training (70%), validation (15%), and test (15%) sets at the patient level. Each patient’s data were grouped into multiple ‘bags’ based on the number of CMR slices available, enabling the use of the MIL framework. To ensure robust evaluation, we employed five-fold cross-validation, where the data set was split into five distinct folds, each with different random seeds. In each fold, the model was trained on the training set, hyperparameter tuning was performed exclusively on the validation set, and the optimized model was then evaluated on an independent test set. The reported results represent the average performance across five independent test sets, ensuring an unbiased assessment of model generalization. A flowchart of the data partitioning and evaluation process is provided in Supplementary material online, *Figure S2*. The model made predictions at the patient level by taking the maximum of all the bag-level predictions, all results presented in the paper were computed at the patient level. To provide additional insights, bag-level results are included in the supplementary material (Supplementary material online, *Figures S3* and *S4*). For evaluation, the area under the receiver operating characteristic curve (AUC-ROC) with a 95% confidence interval (CI) served as the primary performance metric, along with calibration plots. Sensitivity, specificity, precision, Brier score, and *F*-score were calculated across different thresholds. To enhance interpretability, attention scores were analysed to identify and highlight the specific slices that the model focused on, offering valuable insights into its decision-making process. Mean performance metrics, along with 95% CI, were reported to ensure a robust evaluation.

## Results

Of the total 6293 CCTA examinations during the study period, 900 cases had pre-generated CMR images and were included in the study, comprised of 776 cases for the LAD, 694 cases for the RCA, and 600 cases for the LCX. In total, ∼185 500 CMR images were collected. The number of images per CMR reconstruction varied significantly, ranging from 6 to 144 images per case. The patient data were divided into bags of 36 images each, resulting in 1256 bags for LAD, 1241 bags for RCA, and 854 bags for LCX. Refer to *Table 1* for information regarding the presence of stenoses and their distribution across the training, validation, and test sets (at patient level).

**Table 1:** Final population distribution

	LAD	RCA	LCX
*n* (total cases evaluated)	776	694	600
Prevalence of >50% stenosis (%)			
Overall (%)	40.12% (311)	18.75% (130)	15.52% (93)
Training set (70%)	42.21% (217)	18.76% (91)	15.51% (65)
Testing set (15%)	40.17% (47)	19.04% 20)	15.55% (14)
Validation set (15%)	40.17% (47)	19.04% (20)	15.55% (14)
Patient characteristics			
Men, *n* (%)	428 (55.2%)	374 (53.9%)	321 (53.5%)
Women, *n* (%)	346 (44.6%)	319 (46.0%)	278 (46.3%)
Age, mean (SD)	60.9 (13.0)	60.4 (13.3)	60.6 (13.5)

Description of data set used in the classification task, detailing the proportion (in %) and the absolute number of patients (reported in parentheses) belonging to the positive class.

Supplementary material online, *Figure S5* provides detailed information on the distribution of CMR image counts across patients, showing the number of slices generated per patient in various ranges (e.g. 6–10, 10–30) for more specific analysis.

To reproducibility and transparency, we have made the code for pre-processing and model implementation publicly available. The repository includes all relevant scripts and documentation needed to replicate our analyses. It can be accessed at GitHub link: https://github.com/Vibha190685/Multi-Instance-Learning-with-Attention-Mechanism-for-Coronary-Artery-Stenosis-Detection-/tree/main.

### Discriminatory ability

**Figure 3:**
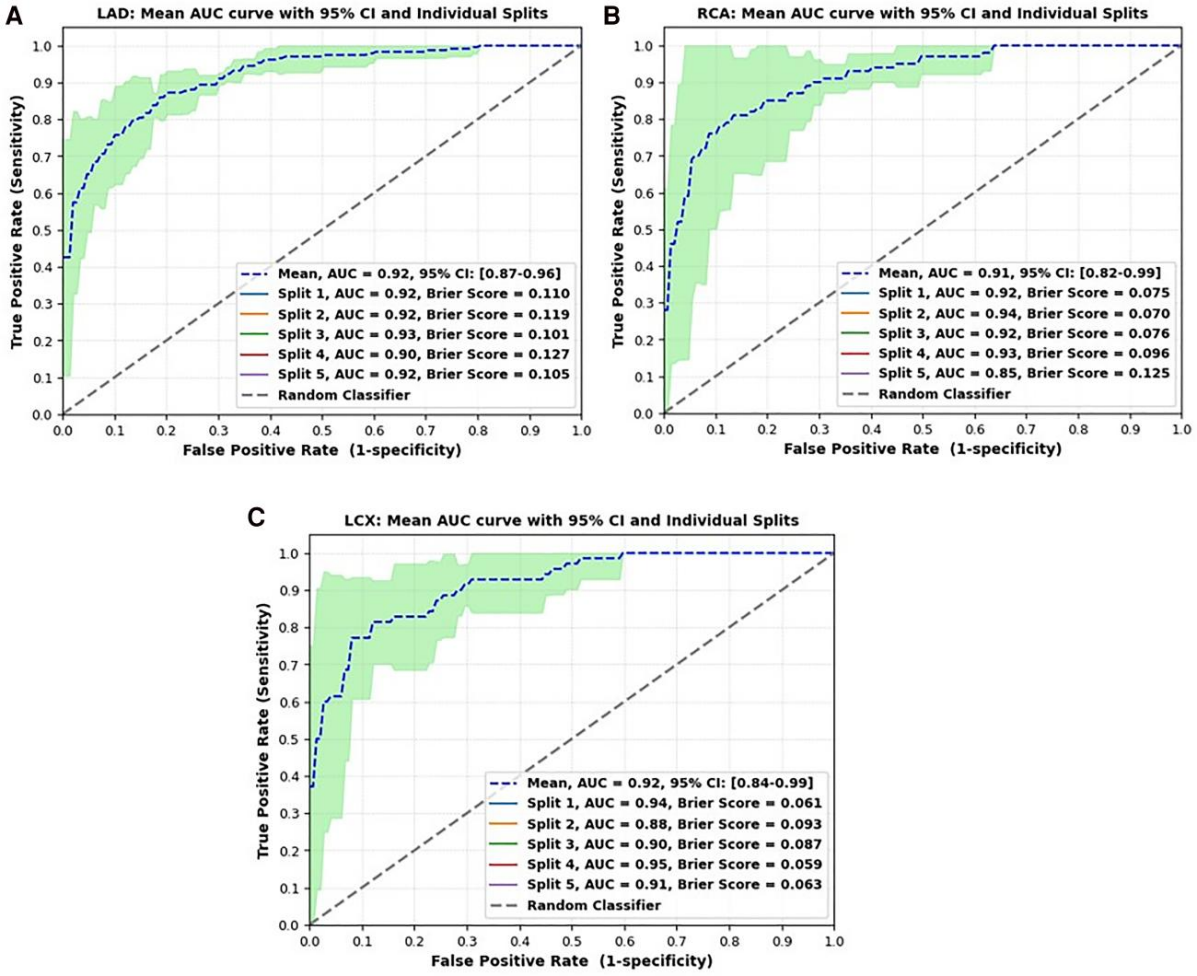
Area under the receiver operating characteristic curve plot illustrating the model’s performance in distinguishing cases with stenosis >50% for left anterior descending, right coronary artery, and left circumflex, along with their 95% confidence intervals (based on test data).

*Figure 3* depicts the AUC-ROC plots for each artery. From *Figure 3A*, it is noted that the model for LAD achieved a mean AUC of 0.92 (95% CI: 0.87–0.96), indicating a high ability to differentiate between cases with stenosis ≥50% and those with stenosis <50%. As presented in *Figure 3B*, the model for RCA achieved a mean AUC of 0.91 (95% CI: 0.82–0.99), while the model for LCX achieved a mean AUC of 0.92 (95% CI: 0.84–0.99) (*Figure 3C*). The mean Brier scores were 0.11 for LAD, 0.09 for RCA, and 0.07 for LCX, reflecting the calibration of predicted probabilities.

### Calibration performance

**Figure 4:**
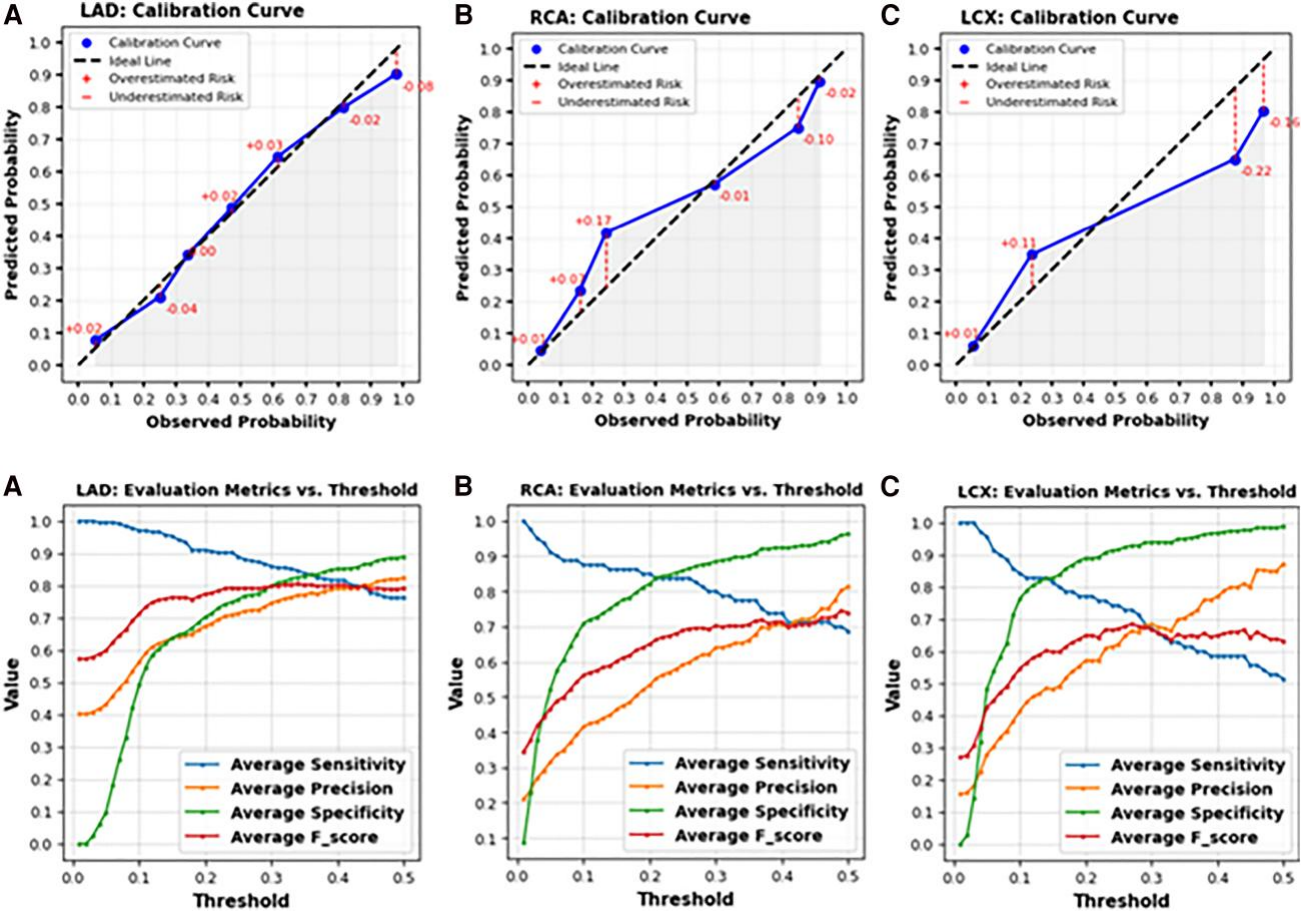
(Top row) Calibration plot illustrating the alignment between predicted probabilities and actual outcomes for cases with stenosis exceeding 50% across. (Bottom row) Analysis of threshold-dependent metric behaviour, illustrating how a change in threshold impacts sensitivity, specificity, and overall predictive performance.

*Figure 4* (top row) presents the calibration plots for LAD, RCA, and LCX. Calibration represents patient-level predictions rather than bag-level, thereby reflecting the performance for the entire case. Left anterior descending exhibited good calibration. The calibration for RCA was inferior to that of the LAD model, with a tendency to overestimate probability in the lower range and underestimate probability in the higher range. The same was true for LCX, although a more pronounced deviation was noted. The lower number of calibration bins for LCX is explained by the presence of fewer positive cases for LCX in the test set.

### Threshold-dependent metrics

Sensitivity, precision, *F*-score, and specificity were analysed across a range of probability thresholds to provide a detailed understanding of the model’s performance across decision thresholds, ranging from 0.01 to 0.5 probability. *Figure 4* (bottom row) displays these plots for LAD, RCA, and LCX. It is evident that for LAD, even at low thresholds (0.2 [20%]–0.3 [30%]), sensitivity reached 90%. Precision and specificity increased from 0.7 to 0.8. Furthermore, as decision thresholds increased up to 0.45, sensitivity remained relatively stable around 80. This suggests that adjusting the threshold within this range can significantly impact these metrics, particularly precision and specificity, without substantially affecting sensitivity and negative predictive value. In contrast, both RCA and LCX exhibited poorer performance in terms of sensitivity compared with LAD, which may be attributed to the lower number of positive cases available for these arteries.

### Attention score analysis

**Figure 5:**
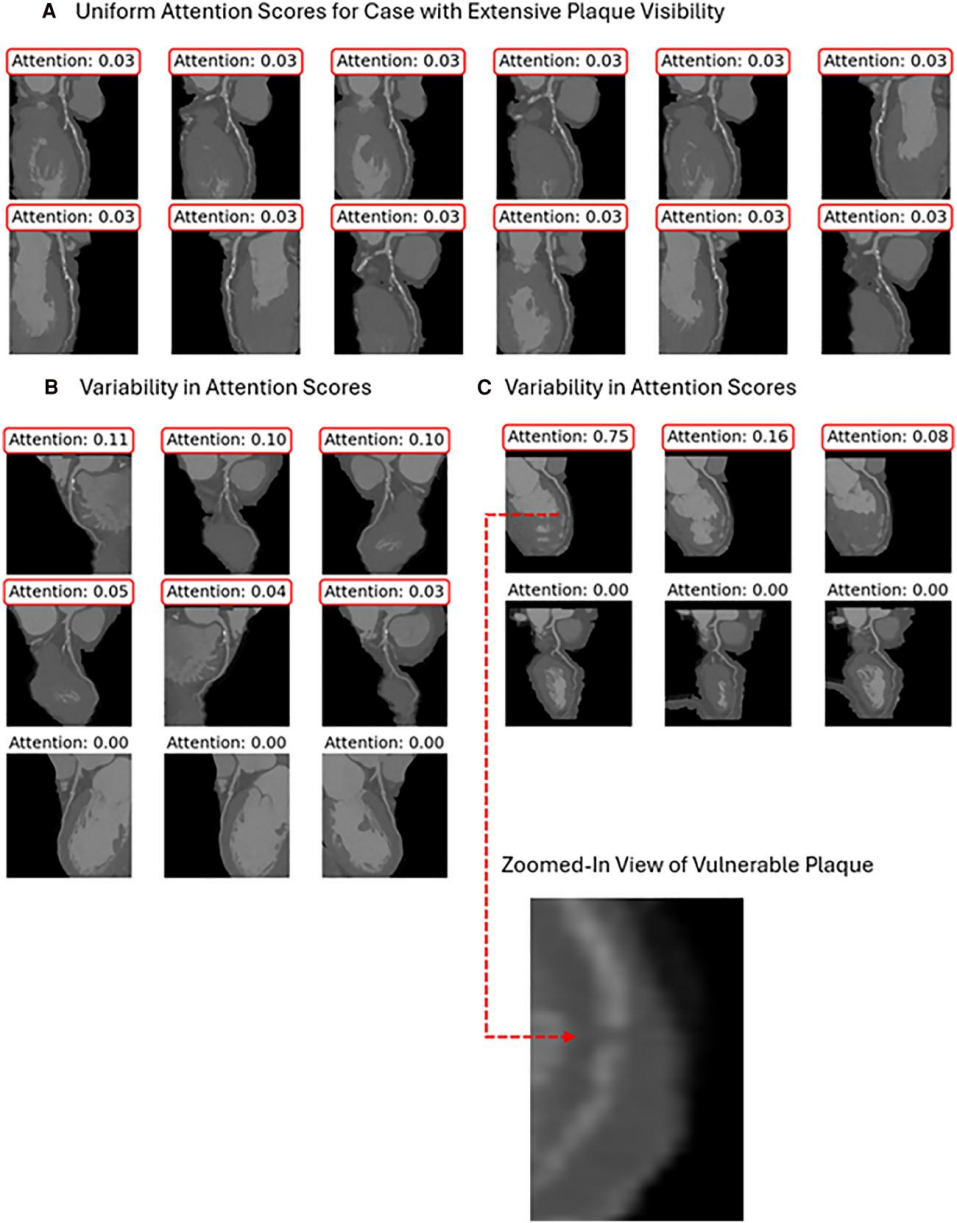
Analysis of attention scores across different cases. (*A*) shows uniform plaque visibility, with evenly distributed attention scores. (*B*) and (*C*) illustrate cases with localized vulnerable plaques, where the model prioritizes slices with clear diagnostic features. A zoomed-in view in (*C*) highlights these regions.

Attention scores were analysed to interpret slice contributions to the final diagnosis, enhancing model transparency and clinical applicability. *Figure 5* illustrates two scenarios: (*Figure 5A*) cases with uniform plaque visibility across slices, where the model assigns equal importance to all slices, indicating consistent recognition when plaque is broadly visible, and (*Figure 5B* and  *C*) cases with localized calcified or vulnerable20 plaques. In these cases, the model prioritizes slices with clear diagnostic features, such as prominent plaques, assigning high attention scores, while slices with indistinct or irrelevant patterns receive lower or zero scores. A zoomed-in view of the vulnerable plaque in *Figure 5C* offers clarity on why the model assigned higher scores to it. Attention scores, normalized to 1 per case, ensure that the distribution of scores across all slices per patient reflects the model’s robust capacity to discern critical features for interpretable coronary artery stenosis detection.

## Discussion

This study highlights the significant potential of utilizing a MIL model to improve the detection of coronary artery stenoses using readily available CMR slices. While CCTA may offer a cost-effective and clinically accurate method for detecting coronary stenoses, its impact on reducing morbidity and mortality associated with CAD is yet to be fully established, especially in primary prevention. Our findings demonstrate that a MIL model can be effectively trained on a relatively small data set to achieve highly accurate predictions while providing slice-level transparency in its decision-making process. For the LAD artery, the model achieved a mean AUC of 0.92, delivering high sensitivity with a low false-positive rate. Additionally, excellent calibration for the LAD artery indicates that the model reliably estimates the presence of stenoses without over- or underestimation, across the range of likelihoods. The mean Brier score of 0.11 further underscores this robustness. This indicates that a model like this can be applied in the clinic both with and without machine dichotomization.

Given the smaller number of cases for the RCA and LCX compared with the LAD, we anticipated that these models would perform inferiorly, a hypothesis that was confirmed. Despite this, the model for the RCA achieved a mean AUC of 0.91, albeit with a CI of 0.82–0.99. The mean Brier score for the RCA was 0.09, indicating reliable predictions but highlighting room for improvement. Although specificity for the RCA exceeds 90%, sensitivity fluctuates between 0.80 and 0.60 across thresholds from 0.2 to 0.5, suggesting that prioritizing high specificity may compromise sensitivity in certain scenarios. Similarly, the LCX model demonstrated a mean AUC of 0.92; although this performance is tempered by sensitivity limitations due to the low prevalence of positive cases, with only 16% of cases being positive for LCX. The mean Brier score for LCX was 0.07, indicating reliable predictions with minimal discrepancies between predicted probabilities and actual outcomes.

The narrower CI s for the LAD (95% CI for AUC-ROC: 0.87–0.96) indicated a more reliable and consistent performance, but the varying anatomy of the LCX, combined with the limited number of positive cases for both the LCX and RCA, may have hampered the model’s predictive ability for these arteries. This highlights the importance of considering anatomical variability and data availability when evaluating model performance. Addressing the low prevalence of positive cases and accounting for anatomical differences in the RCA and LCX could improve the model’s accuracy and its capacity to generalize across diverse clinical scenarios.

The interpretability of the model was qualitatively assessed through attention scores, which highlight the contribution of individual slices to the diagnostic process. As demonstrated by the attention scores in *Figure 5*, our models showed strong proficiency in identifying both calcified plaques and low-attenuating (soft) plaques. Recognizing soft plaques is particularly critical, as they are more vulnerable to triggering acute coronary syndromes.^14,20^ By identifying slices corresponding to high attention scores, the model allowed for targeted analysis of diagnostically critical slices, potentially reducing the number of slices a clinician needs to review to make a diagnosis. This approach may streamline the diagnostic workflow, focusing efforts on slices where the model has observed critical features, thereby saving time and improving efficiency in clinical decision-making. The ability of machine learning models to provide explainability and transparency will be crucial for the foreseeable future, as these models become integrated into clinical workflows as tools to augment decision-making. Explainable outputs, such as attention maps, help build trust, enhance usability, and ensure that clinicians understand how the model arrives at its predictions, which is critical in high-stakes decisions like those needed in cardiovascular medicine.

When compared with existing studies, the CNN-CASS study^21^ achieved an 80% patient-level accuracy using a Shuffle Net V2-based approach on a fixed 50-image stack, which consisted of uniformly generated slices common across all patients. Our model, however, benefits from a more diverse data set of ∼900 patients, accommodating a variable number of slices per case. Other studies, including studies by Penso *et al*., Bian *et al*., and Zreik *et al*., also utilized consistent MPR data from standardized protocols, leading to equal slice counts per patient and demonstrating impressive performance.^20,22–24^ For instance, Penso *et al*. achieved high sensitivity for stenosis detection using a token-mixer architecture (ConvMixer), while Bian *et al*. reached impressive accuracy (88%) and specificity (92%) with a transformer network and self-supervised learning on only 78 patients. Zreik *et al*. employed a multi-task recurrent convolutional neural network on 163 scans, attaining segment-level, artery-level, and patient-level accuracies of 0.94, 0.93, and 0.85, respectively. In contrast, our real-world data were collected across multiple hospitals at different times and by various personnel, introducing real-world variability in most aspects of CCTA. While performance metrics may not be directly comparable, our results align well with existing literature and are based on a more diverse data set, enhancing their external applicability. To provide a clearer comparison, we summarize the AUROC values from similar studies in Supplementary material online, *Table S1*. However, direct statistical comparisons (e.g. DeLong test) were not feasible due to the lack of required metrics in these studies.

This study utilized a binary classification approach, primarily driven by the clinical context where the focus is often on detecting significant stenosis thresholds rather than the precise degree of narrowing, which might require a more nuanced multi-class categorization. Additionally, the data set available for training did not provide sufficient examples across all categories for a multi-class model. Therefore, a binary classification approach was more feasible and practical at this stage of development. Despite the promising results obtained in this study, several limitations must be acknowledged. Primarily, the MIL approach is inherently data-hungry, requiring a larger data set for optimal performance. While our findings are encouraging, we anticipate that expanding our training set to include a more extensive database, as well as incorporating emerging modalities such as fractional flow reserve, plaque characterization, and fat attenuation index for assessing perivascular inflammation, will further enhance model performance and risk prediction. An expanded data set will enable future studies to utilize a multi-class classification approach, allowing for a deeper analysis and more precise differentiation of varying degrees of stenosis. Variability in CMR slice quality remains a challenge. While some slices provided clear, centrally located arterial structures, others were obstructed by surrounding tissues, poorly positioned, or inconsistently bright, ranging from overexposed slices that lost critical detail to darker, noisier ones. These inconsistencies likely affected the attention mechanism and highlight the need for robust image normalization and standardized acquisition protocols to optimize machine learning workflows. Furthermore, the reliance on ground-truth labels derived from radiologist reports of CCTA, which are subject to interobserver variability and inherent limitations of CT imaging in assessing coronary artery stenoses, yields a model which is trained on suboptimal labels. Challenges such as evaluating calcified plaques or subtler stenoses could introduce uncertainty into the model’s performance assessment. Nonetheless, this study underscores the potential of MIL to provide meaningful insights and support clinical decision-making even in the face of variability in ground-truth data.

To enhance the clinical applicability and trustworthiness of our approach, future research should focus on validating the model against gold-standard diagnostic techniques, such as invasive coronary angiography, or adopting consensus-based labelling for greater reliability. Furthermore, analysing attention scores and their variability in greater detail will be pivotal for building confidence in the model’s interpretability and reliability, facilitating its integration into clinical workflows. Refining the model to better address variability in slice quality and expanding the data set to include more diverse and consistent cases will be key steps towards improving both performance and trust in clinical setups.

## Conclusions

This study demonstrates the potential of MIL models to enhance the accuracy of CAD assessments through advanced image analysis. By leveraging innovative modelling techniques and robust pre-processing, we have emphasized relevant features, resulting in improved diagnostic performance. The model’s end-to-end architecture and interpretability not only align with real-world clinical needs but also highlight its potential for future enhancements as data sets expand and undergo further validation. Such advancements are crucial for the widespread adoption of an artificial intelligence-driven solutions in cardiac imaging, ultimately contributing to better patient management and treatment outcomes.

## Supplementary Material

ztaf029_Supplementary_Data

## Data Availability

Data cannot be shared publicly due to ethical and privacy considerations. They include sensitive patient information and disease diagnoses specific to the hospital setting.
