# Supplementary material for: Multi-instance learning with attention mechanism for coronary artery stenosis detection on coronary computed tomography angiography
Source: Eur Heart J Digit Health. 2025 Apr 1;6(3):382–91. doi: 10.1093/ehjdh/ztaf029 (PMC12088718; doi:10.1093/ehjdh/ztaf029)
Supplement: ztaf029_Supplementary_Data [file ztaf029_supplementary_data.docx]

**Supplemental File For Peer Review**

Supplementary Figures and Tabel


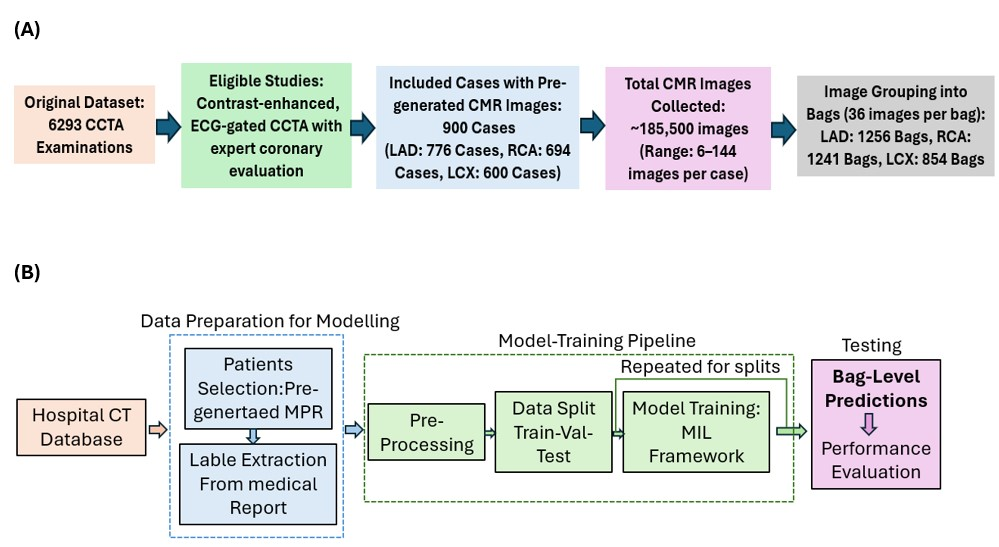


Supplementary Figure 1: Study Design Overview (A) Flowchart detailing the patient recruitment process, including selection criteria and participant flow into the study. (B) Schematic representation of the modelling workflow, encompassing data acquisition, preprocessing, splitting, and evaluation procedures.


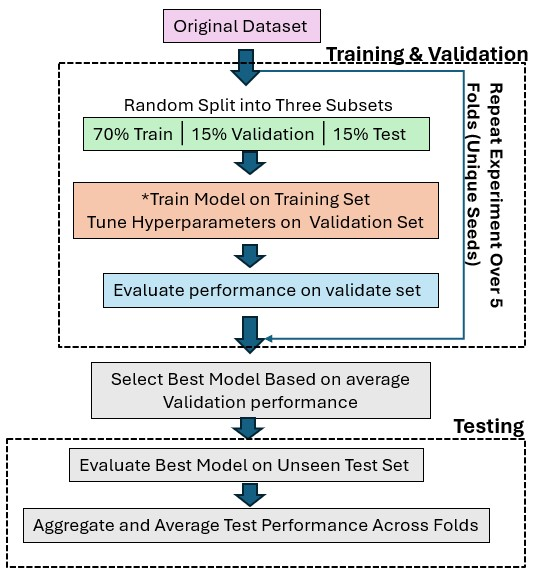


Supplementary Figure 2: Flowchart- Data Splitting and Evaluation Process.


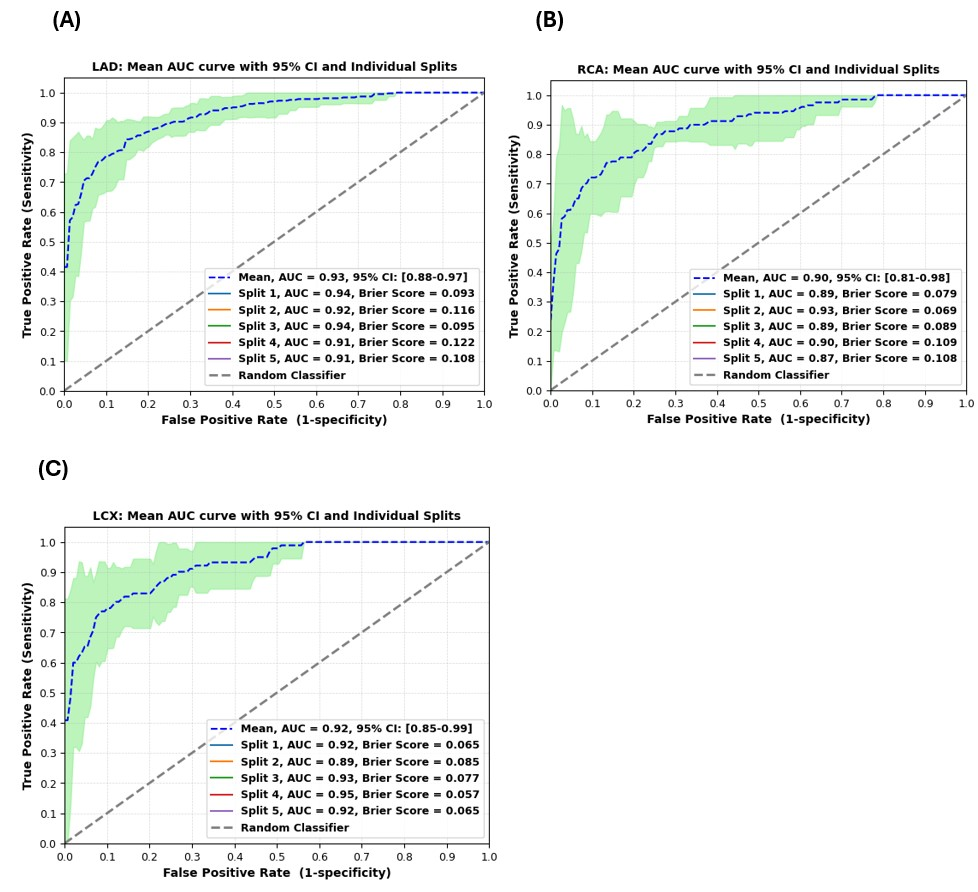


Supplementary Figure 3: Bag-level-prediction- AUC-ROC plot illustrating the model's performance in distinguishing cases with stenosis greater than 50% for LAD, RCA, and LCX, along with their 95% confidence intervals (on test data).


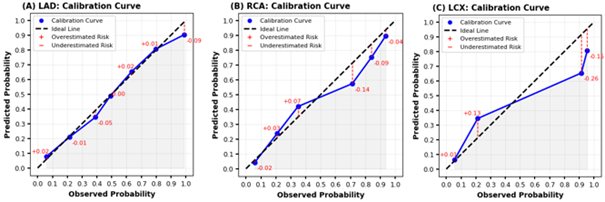


Supplementary Figure 4: Bag-level-prediction, Calibration plots.


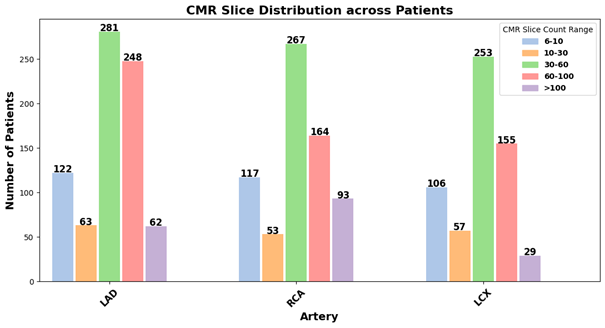


Supplementary Figure 5: CMR images count distribution across patients.

Supplementary Table 1: Comparison of Coronary Artery Disease Classification Performance Across Studies

| **Study** | **LAD (ROC AUC, 95% CI, n)** | **LCX (ROC AUC, 95% CI, n)** | **RCA (ROC AUC, 95% CI, n)** | **Overall Performance Metrics** |
| --- | --- | --- | --- | --- |
| **Current Study (Gupta et al.)** | 0.92 (0.87–0.96), 776 | 0.91 (0.82–0.99), 694 | 0.92 (0.84–0.99), 600 | - |
| **Dobko et al. (2020) [21]** | NA, 824 | NA, 722 | NA, 721 | Overall Accuracy: 0.81, F1-score: 0.82 |
| **Penso et al. (2023) [22]** | NA, NA | NA, NA | NA, NA | Overall AUROC: 0.93 (0.87–0.98), Total Patients: 288 |
| **Bian et al. (2022) [23]** | NA, NA | NA, NA | NA, NA | ACC: 0.8894, Sens: 0.7908, Prec: 0.7901, Spec: 0.9247, F1: 0.7904, MCC: 0.7153 |
| **Zreik et al. (2019) [24]** | NA, 528 (seg) | NA, 426 (seg) | NA, 305 (seg) | ACC: 0.8500, Sens: 0.7223, Prec: 0.7130, Spec: 0.8957, F1: 0.7176, MCC: 0.6154 |

Table legend: Comparison of the performance of our AI-based CAD-RADS classification model with previous studies. The table presents the ROC AUC values with confidence intervals (CI) for the left anterior descending (LAD), left circumflex (LCX), and right coronary artery (RCA), where available. Overall accuracy, sensitivity (Sens), precision (Prec), specificity (Spec), F1-score (F1), and Matthews Correlation Coefficient (MCC) are also reported. "NA" indicates that the value was not provided in the original study. Studies marked with "(seg)" report segment-level evaluations rather than per-patient metrics. "N" refers to the total number of cases.
